# Dam‐Induced Displacement and Disruption Are Associated With Salivary Cortisol Concentration and Patterns of Diurnal Variation

**DOI:** 10.1002/ajhb.70296

**Published:** 2026-06-22

**Authors:** Cassie C. Lee, Aaron A. Miller, Thomas W. McDade, Patrick M. Owuor

**Affiliations:** ^1^ Department of Anthropology Northwestern University Evanston Illinois USA; ^2^ Institute for Policy Research Northwestern University Evanston Illinois USA; ^3^ Department of Anthropology Wayne State University Detroit Michigan USA

**Keywords:** Infrastructure development, Salivary cortisol, Thwake Multipurpose Dam

## Abstract

**Objectives:**

This study assesses the stress‐related impacts of the construction of the Thwake Multipurpose Dam in Makueni, Kenya by examining salivary cortisol concentrations and patterns of diurnal variation.

**Methods:**

One set of evening, waking, and 30‐min post‐waking saliva samples was collected across 221 women who were displaced by the dam or who lived upstream or downstream of the dam development site. Salivary cortisol concentration was analyzed using a commercially available assay kit. Multivariable linear regression was used to assess the relationship between displacement status and waking cortisol concentration, evening cortisol concentration, cortisol awakening response, and diurnal difference.

**Results:**

Log‐transformed evening cortisol concentration (displaced: *β* = 0.365, *p* = 0.018; downstream: *β* = 0.675, *p* = 0.007) and diurnal difference (displaced: *β* = 0.034, *p* = 0.049) were significantly associated with displacement status.

**Conclusions:**

Both displaced and downstream communities demonstrate stress‐related hormonal differences associated with dam‐induced disruption. Future policy and research addressing the health impacts of hydroelectric dam development should include downstream communities in addition to those directly displaced by development.

## 1 Introduction

Hydroelectric dams are an integral tool for social and economic development (Kumar et al. 2011). However, dam development projects can be socially and ecologically disruptive to nearby communities (Castro‐Diaz et al. 2023; Gracey and Verones 2016; Okuku et al. 2016; Owuor et al. 2023). Further, the disruption associated with such infrastructure projects may have long‐term population health consequences that are not adequately captured by environmental and social impact assessments (Okuku et al. 2016).

Upon completion of construction beginning in 2018, the Thwake Multipurpose Dam in Makueni County, Kenya, is expected to generate electricity, address water insecurity, and boost socio‐economic opportunities in the region (African Development Bank 2013). While previous research examining C‐reactive protein as a marker of systemic inflammation in this setting showed no significant difference between displaced and non‐displaced women approximately 1 year after dam construction began, salivary cortisol as an indicator of stress has not yet been studied (Owuor 2022). To better understand the health effects of hydroelectric dam construction, we examine the relationship between salivary cortisol levels, diurnal variation patterns, and displacement status among women residing near the Thwake Multipurpose Dam site approximately 1 year after construction began. We hypothesize that women who have been displaced by the construction of the Thwake Dam will have stress‐related hormonal differences compared to women who live upstream or downstream of the development site.

## 2 Materials and Methods

### 2.1 Participants and Study Design

Women who lived in the proposed Thwake Multipurpose Dam project area in Makueni County, Kenya for at least 3 months prior to recruitment were invited to participate in the Thwake Dam Construction Health Assessment Study (Owuor 2022). Written informed consent was obtained from all participants with the approval of the Northwestern University Institutional Review Board (STU00208788), the Amref Ethical and Scientific Review Committee (P574/2019), and the National Commission for Science Technology and Innovation research license (584132) and with authorization from the Kenya Ministry of Water and Sanitation. Self‐administered survey data, salivary cortisol, and anthropometric measurements were collected between July and August 2019. Participants were classified into displacement statuses, including displaced individuals, those living downstream, and those living upstream (Comparison) of the Thwake Multipurpose Dam site.

### 2.2 Collection of Saliva and Measurement of Salivary Cortisol Concentration

Participants were asked to collect one set of saliva samples across three time points using the passive drool method in the evening, upon waking, and 30‐min after waking for a total of three saliva samples. Samples were stored at −20°C at the Kenya Medical Research Institute laboratory before being shipped and stored at −80°C at the Laboratory for Human Biology Research at Northwestern University.

Salivary cortisol concentrations were analyzed in duplicate from 1205 samples across 411 participants using a commercially available kit (Salimetrics Assay #1‐3002). High and low controls were analyzed across all assay plates with inter‐assay coefficients of variation (CV) of 3.054% and 5.489%, respectively. All samples with cortisol concentration ≥ 0.030 μg/dL were re‐analyzed if the CV was > 9.999%.

Consistent with prior work demonstrating the impact of noncompliance on cortisol diurnal patterns, samples indicating possible noncompliance were excluded from analysis (Kudielka et al. 2003). An error of ±0.060 μg/dL was used for the exclusion criteria to align with the methods used to identify samples to re‐analyze in the assay and to account for true flat or negligible CAR and diurnal differences. Diurnal difference was operationalized as the difference in the concentration of cortisol between the evening and the waking samples. Participants with CAR ≤ −0.06 μg/dL or diurnal difference ≥ 0.06 μg/dL were excluded from analysis, as a decrease in cortisol concentrations after waking or an increase in cortisol concentration between waking and evening samples may indicate noncompliance. The exclusion of participants was randomly distributed across displacement status (*X* ^2^ = 0.583, df = 2, *p* = 0.747). Additionally, 13 samples identified as potentially bloody, just water, or too acidic and 33 participants with potential inaccuracies in survey data (age, sex, or duplicate responses) were removed from analysis. A total of 221 participants were included in this analysis.

### 2.3 Covariates and Data Analysis

Data were analyzed using R 4.5.2. Observations of waking cortisol concentration, evening cortisol concentration, CAR, and diurnal difference ±3 SD from the mean were removed from analysis (Adam and Kumari 2009). Evening cortisol concentrations were log‐transformed for normality, with concentrations undiscernible from 0.000 μg/dL recoded as 0.001 μg/dL prior to transformation.

Multivariable linear regression models were used to assess the relationship between waking cortisol concentration, evening cortisol concentration, CAR, and diurnal difference and displacement status. Age, BMI, domestic violence score, dietary diversity, household wealth, and perceived stress served as covariates. The Hurt, Insulted, Threatened with harm, and Screamed at (HITS) Scale was used to assess domestic violence (Sherin et al. 1998). Dietary diversity was assessed using the Minimum Dietary Diversity for Women indicator (FAO and FHI 360 2016). Perceived stress was assessed using the 10‐item Perceived Stress Scale (Cohen and Williamson 1988). The first principal component from principal component analysis using participant asset ownership was used to calculate a continuous household wealth index across all individuals included in the Thwake Dam Construction Health Assessment Study (Owuor 2022).

## 3 Results

Of the 221 participants, 79 (35.7%) were displaced, 27 (12.2%) lived downstream, and 115 (52.0%) lived upstream to the Thwake Multipurpose Dam development site. The mean values for age were 37.1 years (SD = 15.7), BMI was 24.6 kg/m^2^ (SD = 5.35), HITS score was 0.260 (SD = 0.819), Minimum Dietary Diversity for Women indicator was 5.72 (SD = 2.01), Perceived Stress Score was 18.4 (SD = 5.92), and wealth index was 0.0700 (SD = 0.901).

**FIGURE 1:**
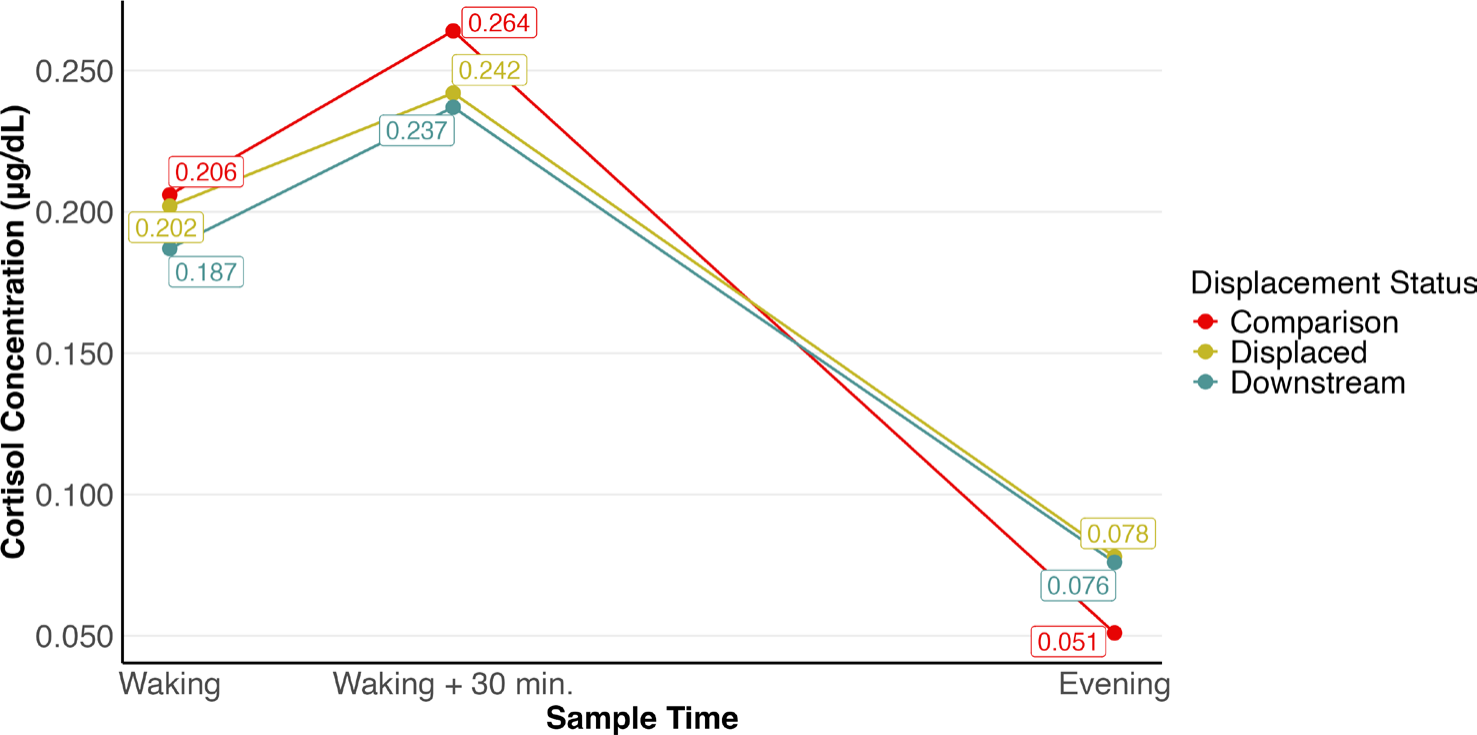
Line graph of cortisol diurnal pattern, stratified by displacement status. Points indicate the mean cortisol concentration per group at each time point. Evening values reflect the prior evening. Across the comparison group (*N* = 115), the mean values for cortisol concentrations were 0.206 μg/dL (SD = 0.109) at waking, 0.264 μg/dL (SD = 0.145) 30‐min post‐waking, and 0.051 μg/dL (SD = 0.044) in the evening. Across the displaced group (*N* = 79), the mean values for cortisol concentrations were 0.202 μg/dL (SD = 0.102) at waking, 0.242 μg/dL (SD = 0.136) 30‐min post‐waking, and 0.078 μg/dL (SD = 0.073) in the evening. Across the downstream group (*N* = 27), the mean values for cortisol concentrations were 0.187 μg/dL (SD = 0.093) at waking, 0.237 μg/dL (SD = 0.114) 30‐min post‐waking, and 0.076 μg/dL (SD = 0.059) in the evening.

Across all participants, the mean values for cortisol concentrations were 0.202 μg/dL (SD = 0.105) at waking, 0.252 μg/dL (SD = 0.138) 30‐min post‐waking, and 0.064 μg/dL (SD = 0.059) in the evening. Figure 1 shows the mean concentrations stratified by displacement status. The mean CAR across all participants was 0.046 μg/dL (SD = 0.079), and the mean diurnal difference was −0.135 μg/dL (SD = 0.110).

In adjusted linear regressions, log‐transformed evening cortisol concentration and diurnal difference were significantly associated with displacement status. Relative to the comparison group, both the downstream and displaced groups had higher log‐evening cortisol concentrations, and the displaced group had less diurnal difference (Table 1).

**TABLE 1:** Adjusted linear regression models for waking cortisol concentration, log‐transformed evening cortisol concentration, cortisol awakening response, and diurnal difference among women who were displaced, those living downstream, and those living upstream of the Thwake Multipurpose Dam site.

Characteristic	Waking cortisol (*N* =204)			Log evening cortisol (*N* = 197)			Cortisol awakening response (*N* = 194)			Diurnal difference (*N* = 195)		
	Beta	95% CI	*p*	Beta	95% CI	*p*	Beta	95% CI	*p*	Beta	95% CI	*p*
Displacement status												
Comparison	—	—		—	—		—	—		—	—	
Displaced	−0.002	[−0.034, 0.031]	0.93	0.365*	[0.062, 0.668]	0.018	−0.017	[−0.042, 0.009]	0.2	0.034*	[0.000, 0.068]	0.049
Downstream	−0.012	[−0.062, 0.038]	0.64	0.675**	[0.188, 1.162]	0.007	−0.024	[−0.064, 0.015]	0.22	0.053	[−0.001, 0.108]	0.055
Age	0.000	[−0.001, 0.001]	0.67	0.008	[−0.001, 0.017]	0.10	0.000	[−0.001, 0.001]	0.79	0.000	[−0.001, 0.001]	0.98
BMI	−0.001	[−0.004, 0.002]	0.51	−0.011	[−0.037, 0.015]	0.40	−0.001	[−0.003, 0.002]	0.61	0.000	[−0.003, 0.003]	0.87
Domestic violence	0.005	[−0.013, 0.024]	0.58	0.018	[−0.160, 0.195]	0.85	0.002	[−0.013, 0.017]	0.78	−0.001	[−0.021, 0.018]	0.88
Dietary diversity	0.000	[−0.008, 0.008]	0.95	−0.045	[−0.122, 0.032]	0.25	0.003	[−0.004, 0.009]	0.38	−0.003	[−0.011, 0.006]	0.54
Perceived stress	0.000	[−0.003, 0.002]	0.78	−0.022	[−0.046, 0.003]	0.08	0.000	[−0.003, 0.002]	0.64	−0.001	[−0.004, 0.002]	0.57
Wealth Index	0.000	[−0.018, 0.017]	0.97	0.004	[−0.157, 0.165]	0.96	0.007	[−0.006, 0.020]	0.31	0.002	[−0.016, 0.020]	0.85

*Note:* *p < 0.05; **p < 0.01; ***p < 0.001.

Abbreviation: CI = confidence interval.

## 4 Discussion

Both women displaced by dam construction and women living downstream of the construction site showed stress‐related hormonal differences compared to women living upstream of construction. Women displaced by the dam had higher levels of evening cortisol concentration and a marginally significant decrease in the magnitude of diurnal difference. Further, women living downstream from the development site also had higher evening cortisol levels.

Lower evening cortisol concentrations and decreased magnitudes of diurnal difference contribute to and are indicative of flatter cortisol diurnal slopes (Adam et al. 2017; Adam and Kumari 2009). These results indicate that the stress of disrupted livelihoods becomes embodied in both displaced women and those living downstream of the dam. This is consistent with prior work demonstrating that economic, environmental, and psychosocial stressors were experienced by both displaced and non‐displaced women living in and around the Thwake Dam development area (Owuor et al. 2023). However, these results contrast with previous analysis showing no difference in systemic inflammation between displaced and non‐displaced women in this setting (Owuor 2022). Because C‐reactive protein concentrations and cortisol diurnal patterns were assessed using samples collected at the same time point, these analyses suggest that salivary cortisol may be an earlier or more sensitive indicator of the biosocial impacts of stress than C‐reactive protein in this population, particularly when a stressor may not have yet become a chronic stressor (Rohleder 2019). Additionally, prior analysis of the Thwake Dam Environmental and Social Impact Assessment Report and Resettlement Action Plan revealed discrepancies between the anticipated impacts of hydroelectric dam development and the host communities' experiences (Shisoka et al. 2025). To further address these discrepancies, we recommend including downstream communities in future planning.

Limitations of this study include the lack of data regarding sample collection timing. However, due to the sensitivity of cortisol concentration to the time since waking, we were able to use CAR and diurnal difference to exclude possible noncompliance from analysis (Kudielka et al. 2003). Additionally, we were unable to directly assess diurnal decline because evening samples were collected the night before. Nonetheless, we were able to use the difference between evening and waking cortisol concentrations as a proxy for diurnal decline. Further, cortisol concentrations from a single set of daily saliva samples may not represent participants' typical diurnal patterns, and future work should collect saliva samples across multiple days to better assess typical diurnal patterns. Finally, the sample size of the downstream group is relatively small compared to the other groups. However, these results provide evidence for further exploration of the health impacts of hydroelectric dam development on downstream communities.

## 5 Conclusion

These analyses suggest that both women displaced by and living downstream of the dam construction site show stress‐related hormonal differences, as measured by salivary cortisol. We recommend including downstream communities, in addition to those directly displaced by development, in policies and research designed to support communities affected by hydroelectric dam development.

## Author Contributions

**Cassie C. Lee:** conceptualization; analysis, writing – original draft, writing – review and editing. **Aaron A. Miller:** conceptualization, analysis, writing – review and editing. **Thomas W. McDade:** analysis, writing – review and editing. **Patrick M. Owuor:** conceptualization; analysis, data collection; writing – review and editing.

## Funding

This work was supported by Wayne State University Department of Anthropology and Northwestern University Institute for Policy Research.

## Disclosure

AI was not used in the writing, editing, or data analysis of this manuscript.

## Ethics Statement

Written informed consent was obtained from all participants with the approval of the Northwestern University Institutional Review Board (STU00208788), the Amref Ethical and Scientific Review Committee (P574/2019), and the National Commission for Science Technology and Innovation research license (584132), and with authorization from the Kenya Ministry of Water and Sanitation.

## Conflicts of Interest

The authors declare no conflicts of interest.

## Data Availability

The data that support the findings of this study are available from the corresponding author upon reasonable request.
